# Relax, Cool Down and Scaffold: How to Restore Surface Expression of Folding-Deficient Mutant GPCRs and SLC6 Transporters

**DOI:** 10.3390/ijms18112416

**Published:** 2017-11-14

**Authors:** H.M. Mazhar Asjad, Shahrooz Nasrollahi-Shirazi, Sonja Sucic, Michael Freissmuth, Christian Nanoff

**Affiliations:** Institute of Pharmacology and the Gaston H. Glock Research Laboratories for Exploratory Drug Development, Center of Physiology and Pharmacology, Medical University of Vienna, A-1090 Vienna, Austria; n1442638@students.meduniwien.ac.at (H.M.M.A.); shahrooz.nasrollahishirazi@meduniwien.ac.at (S.N.-S.); sonja.sucic@meduniwien.ac.at (S.S.); christian.nanoff@meduniwien.ac.at (C.N.)

**Keywords:** G protein coupled receptors/GPCRs, solute carrier 6/SLC6, misfolding, heat-shock protein relay, pharmacochaperoning, heat-shock protein inhibitors

## Abstract

Many diseases arise from mutations, which impair protein folding. The study of folding-deficient variants of G protein-coupled receptors and solute carrier 6 (SLC6) transporters has shed light on the folding trajectory, how it is monitored and how misfolding can be remedied. Reducing the temperature lowers the energy barrier between folding intermediates and thereby eliminates stalling along the folding trajectory. For obvious reasons, cooling down is not a therapeutic option. One approach to rescue misfolded variants is to use membrane-permeable orthosteric ligands. Antagonists of GPCRs are—in many instances—effective pharmacochaperones: they restore cell surface expression provided that they enter cells and bind to folding intermediates. Pharmacochaperoning of SLC6 transporters is less readily achieved because the ionic conditions in the endoplasmic reticulum (ER) are not conducive to binding of typical inhibitors. The second approach is to target the heat-shock protein (HSP) relay, which monitors the folding trajectory on the cytosolic side. Importantly, orthosteric ligands and HSP-inhibitors are not mutually exclusive. In fact, pharmacochaperones and HSP-inhibitors can act in an additive or synergistic manner. This was exemplified by rescuing disease-causing, folding-deficient variants of the human dopamine transporters with the HSP70 inhibitor pifithrin-μ and the pharmacochaperone noribogaine in *Drosophila melanogaster*.

## 1. Introduction

Pharmacological chaperoning or pharmacochaperoning refers to the ability of small molecules to increase the expression of their target protein by enhancing productive folding [1,2]. Several pharmacochaperones have recently been approved for clinical use; prominent examples include migalastat and lumacaftor, which restore folding of some mutants of lysosomal α-galactosidase in Fabry’s disease [3] and of CFTR-ΔF508, the most frequently mutated variant of the cystic fibrosis transmembrane conductance regulator (CFTR/ABC-C7) [4]. It has, however, been known for some 40 years that antagonists can enhance the expression of their cognate receptors. This was originally observed in people who had been treated with the β-adrenergic antagonist propranolol: sudden cessation of β-adrenergic receptor blockade resulted in a pronounced increase in angina pectoris attacks and in frequent myocardial infarctions [5,6]. It was subsequently appreciated that this “propranolol withdrawal rebound” was accounted for by elevated β-adrenergic receptor levels at the cell surface in both people [7] and experimental animals [8]. Originally, it was thought that these increase surface levels reflected a reduced internalization rate of the antagonist-bound β-adrenergic receptors. However, it is now clear that the antagonists act in the endoplasmic reticulum (ER); three observations support this conclusion: (i) the pharmacochaperoning action is contingent on the cell permeability of the antagonist. Landiolol, which is hydrophilic and poorly enters cells, fails to increase the cell surface levels of β_1_-adrenergic receptors; in contrast, esmolol and propranolol—two antagonists that readily enter cells—promote cell surface expression of the receptor [9]. The action is specific because the non-selective antagonist exerts its actions on both β_1_- and β_2_-receptors, while the action of the β_1_-selective antagonist is confined to β_1_-receptors [9]; (ii) The pharmacochaperoning action is dependent on a functional ER export machinery: if the formation of COPII (coatomer protein complex II) coat is disrupted by siRNA-dependent depletion of the cargo receptors SEC24A-D, both propranolol and esmolol fail to increase receptor levels at the cell surface [9]; (iii) The antagonist-induced export from the ER can also be directly visualised under the microscope: within an hour after antagonist application, the refolded receptor is delivered to and concentrated in the Golgi apparatus [10]. Based on these and related findings, it is safe to conclude that cell-permeable ligands act on their targets within the secretory pathway to increase the rate of their delivery to the cell surface [11]. 

**Figure 1:**
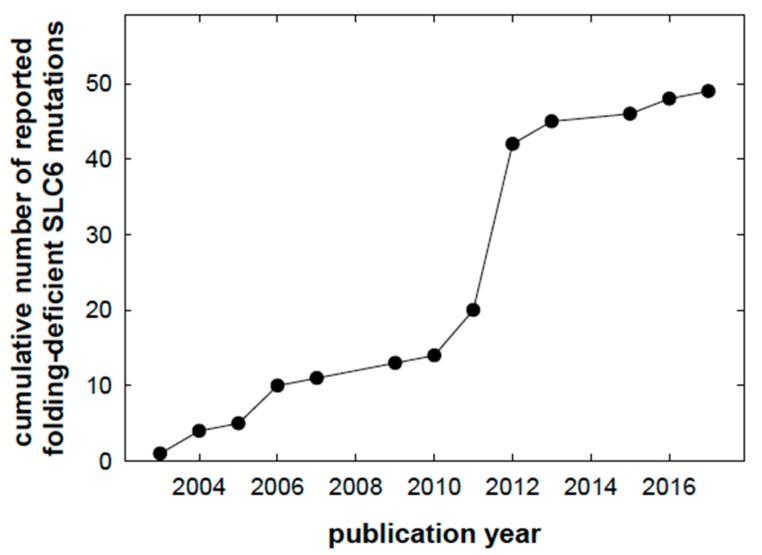
Cumulative number of point mutations in the coding sequence of mutations, which result in folding-deficient solute carriers (SLC) transporters. The publications were identified in PubMed (www.ncbi.nlm.nih.gov). The numbers are a conservative estimate: only coding variants were counted, where the experimental evidence indicated a loss of function due to misfolding. Truncations due to premature stop codons were ignored, as were mutations, which resulted in a disrupted binding site for substrate and co-substrate ions. The pertinent references are for the norepinephrine transport (NET/SLC6A2 [17], for the creatine transporter-1 (CT1/SLC6A8 [18,19,20,21,22,23,24,25,26,27,28]), for the glycine transporter-2 (GlyT2/SLC6A5 [29,30]), for the dopamine transporter (DAT/SLC6A3 [31,32,33]) and for the GABA-transporter-1 (GAT1 [34]).

Sickle cell anemia was the first disease appreciated to result from protein misfolding due to a point mutation (E6V of the globin β-chain). In fact, the term molecular medicine was coined to highlight the paradigm shift that arose from the insights into protein misfolding [12]. Sickle cell anaemia can be considered a highly prevalent monogenic folding disease [13]. In all other instances, individual mutant alleles, which encode a folding-deficient version of a protein, are rare. However, collectively, folding diseases account for a large fraction of monogenic diseases. It is also clear that the currently known number must be an underestimate. G protein-coupled receptors (GPCRs) and solute carriers (SLC) comprise the largest and second largest family of membrane proteins with some 800 and 400 members encoded by the human genome, respectively. Thus, collectively, they account for one-fifth of the roughly 6000 membrane proteins. In folding-deficient mutants of GPCRs, the approach to pharmacochaperoning is—in principle—straightforward: antagonist ligands are predicted to act as pharmacochaperones. This prediction was first verified for misfolded V2-vasopressin receptor mutants, which give rise to diabetes insipidus [14]. Transporters are more challenging (see below). There is a long list of mutations that have been shown to result in misfolding of GPCRs [15]; however, it is clear that this list is not exhaustive, because the number of identified mutants keeps growing. This is also true for transporters of the SLC family. To illustrate the point, we selected those nonsynonymous coding mutations reported in the SLC6 family, which give rise to *bona fide* misfolded proteins. It is evident from the graphic representation in Figure 1 that the cumulative number of disease-associated, folding-deficient mutant has been continuously increasing over the past two decades. Based on this snapshot, it is safe to posit that disease-associated folding-deficient mutants will be identified in each family of membrane proteins. This is also consistent with a large survey covering 1200 human proteins and 2477 disease-associated missense mutations thereof: at least one-third of these result in a folding deficiency [16]. 

## 2. The C-Terminus as a Folding Checkpoint

**Figure 2:**
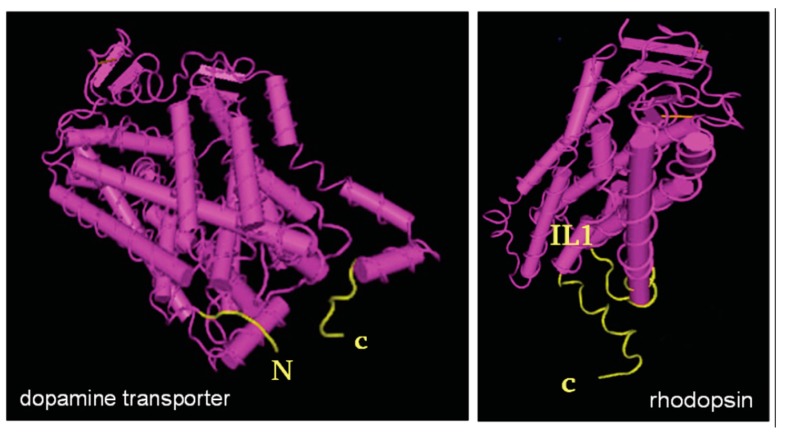
Structures of an SLC transporter (dopamine transporter) and a GPCR (rhodopsin) highlighting the circular arrangement of the helices in hydrophobic core. In spite of the truncations of the N- and C-terminal peptide segments, the structures indicate that juxtamembrane N- and C-terminal portions (highlighted in yellow) meet. In the transporter, the structure suggests an end-to-end contact of the cytoplasmic segments. In the receptor, helix 8 of the cytoplasmic carboxyterminus approaches α-helix 1/cytoplasmic loop 1 (IL1). Either arrangement likely serves as latch to stabilize the circular structure. It is also evident that several helices do not run perpendicular to the plane of the membrane; tilting is a reflection of the hydrophobic mismatch, which imposes an energy barrier during the conformational search associated with folding. View from an intracellular (cytosolic) perspective of the tilted dopamine transporter. Extracellular and lateral view of rhodopsin. Structure models (dopamine transporter 4XP4; rhodopsin 2I36) taken from www.ncbi.nlm.nih.gov.

We should like to argue that properties that are shared among polytopic membrane proteins of distinct classes are likely to reflect general principles. Hence, insights gained from studying a limited number of examples from two distinct classes of polytopic membrane proteins are also likely to have repercussions for many other protein families. GPCRs and SLC6 transporters differ substantially in their topology: GPCRs have seven transmembrane-spanning α-helices (TM1 to TM7) resulting in an extracellular N-terminus and an intracellular C-terminus. The hydrophobic core of SLC6 transporters comprises twelve transmembrane-spanning α-helices (TM1 to TM12). Because of the even number of transmembrane segments, the N- and C-termini must be on the same side of the membrane, in this instance on the cytosolic side. Nevertheless, GPCRs and SLC6 transporters face a similar folding problem: their transmembrane segments are cotranslationally inserted into SEC61 translocon channel and are released into the lipid milieu of the ER membrane via a lateral gate as an individual α-helix or pairwise [35]. However, the helices must adopt an annular arrangement. Thus, membrane lipids must be displaced on one side to allow for helix packing. Conversely, on the side exposed to the lipid bilayer, the acyl-side chains of the membrane lipids must be accommodated by the helices. The resulting hydrophobic mismatch imposes an energy barrier during the folding and rearrangement of helices [36]. It is therefore not surprising that disease-associated, folding-deficient mutants of SLC6 transporters fall into two major classes: they either map to the lipid/protein interface or they are likely to affect helix packing by replacing glycine residues with bulkier side chains [37,38,39]. This is particularly evident for mutants of the dopamine transporter (DAT/SLC6A3) and of the creatine transporter-1 (CrT1/SLC6A8), which are associated with a syndrome of infantile dystonia/Parkinsonism and intellectual disability/mental retardation, respectively. Of the 17 CrT-1 and the 13 DAT mutants, which give rise to a disease due to folding-deficiency, six and three affect intramembrane glycine residues, respectively [38,39]. The helical bundle of the hydrophobic core must be stabilized to prevent lipids from invading the hydrophobic core. Several lines of evidence suggest that this is achieved by the C-terminus in both GPCRs and SLC6 transporters (Figure 2): serial truncations of the C-terminus, for instance, inactivate the A_1_-adenosine receptor such that its hydrophobic core fails to bind ligands [40]. This is also true for SLC6 transporters [41,42,43]. In fact, the C-terminus of the serotonin transporter (SERT/SLC6A4) interacts with the first intracellular loop (IL1) via a salt bridge [44]. Molecular dynamics simulations also highlight the role of the C-terminus in driving the progression of GPCRs to the minimum energy conformation; a large drop in free energy is associated with packing of the proximal segment of the C-terminus against a hydrophobic pocket created between TM1 and TM7 [45]. 

**Figure 3:**
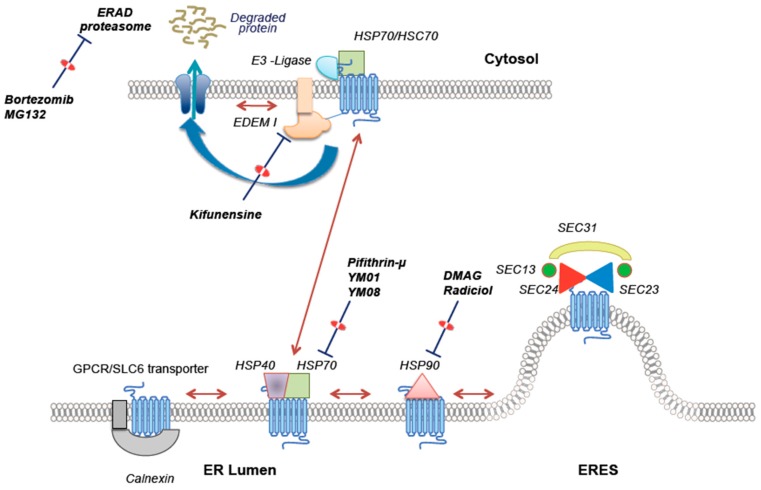
Extended chaperone/COPII-exchange model. Upon release from the SEC61 translocon channel (not shown), the nascent transmembrane protein (GPCR or SLC6 transporter symbolised by blue TM helices) is glycosylated and engaged by calnexin; subsequently a heat-shock protein relay is recruited to the C-terminus with sequential binding of HSP40 and HSP70, (which can be inhibited by pifithin-μ, YM01, YM08, etc.) followed by transfer to HSP90 (which can be inhited by DMAG = 17-(dimethylaminoethylamino)-17-demethoxygeldanamycin = alvespimycin, radiciol and related compounds). If the client protein reaches its stable fold, the heat-shock proteins are released. This licences the C-terminus for an interaction with the cognate SEC24-isoform. The SEC23/SEC24-dimer (bow tie-shaped red and blue triangles) incorporates the protein cargo at ER exit sites (ERES) into the nascent COPII (coatomer protein complex II) coated vesicle, the curvature of which is induced by the bow tie-shape and stabilized by the outer layer COPII components SEC13/SEC31. For the sake of simplicity, the additional co-chaperones of the heat-shock protein relay (HOP, AHA1, etc.) and the additional components of the COPII machinery (the guanine nucleotide exchange factor SEC12 and the G protein SAR1) are not shown. If a stable fold cannot be reached, the protein is eventually marked for ER-associated degradation (ERAD) by recruitment of an E3 ubiquitin ligase. This can be triggered by the cytosolic heat-shock protein relay (shown in the upper part) or by lumenal chaperones (not shown). Initiation of ERAD is also contingent on a kifunensine-inhibited lumenal mannisodase (ER degradation-enhancing alpha-mannosidase-like protein 1—EDEM1). After retrotanslocation, the protein is degraded by the proteasome, which is susceptible to inhibition by MG132, bortezomib and related compounds. Note that for the sake of clarity the series of events have been depicted in two separate schematic representations, but they occur in the same plane of the membrane.

Folding of membrane proteins in the ER is assisted by an array of lumenal chaperones; for GPCRs, the folding trajectory is monitored by general (“public”) chaperones such as calnexin, BiP/GRP78, protein disulphide isomerase and GRP94 [15,43]. In addition, individual GPCRs are assisted by specific (“private”) chaperones, which are required for reaching the folded state and for ER export [15,46], e.g., receptor transporting proteins/RTPs for odorant receptors [47]. This also appears to be the case for some SLC6 transporters: both, the amino acid transporters B°AT3/SLC6A18 and B°AT1/SLCA19 require collectrin or ACE2 (angiotensin-converting enzyme 2) to reach the cell surface [48,49,50]. In addition, the folding trajectory of GPCRs and of SLC6 transporters is also monitored on the cytosolic side. This is accomplished by a heat-shock protein (HSP) relay, which operates—at least in part—on the C-terminus (Figure 3). The evidence is as follows: when heterologously expressed the A_2A_-adenosine receptor accumulates in the endoplasmic reticulum [51,52]. This is not an artefact of heterologous overexpression because it can also be seen in PC12 cells, which express the A_2A_-receptor endogenously [53]. These receptors are stalled along their folding trajectory and have not reached their stable conformation because they accumulate in complexes with calnexin [52,54]. Mass spectrometry of tandem affinity-purified receptors shows that they are not only retrieved in complexes with ER lumenal chaperones but also with abundant amounts of HSP90α and HSP70-1A [52]. In addition, the complex contains typical components of the heat-shock protein relay [52], e.g., HOP (HSC70-HSP90-organizing protein), p23 (HSP90 co-chaperone) and CHIP (C terminus of HSP70-interacting protein, an E3-ubiquitin ligase) [55]. This suggests that the stalled folding intermediates are shuttled back and forth by the heat-shock relay to allow for refolding with two possible eventual outcomes, degradation or ER export. In fact, the flux of protein through this relay can be modified by changing the levels of individual components, e.g., depletion of HSP90α or of CHIP by siRNA-mediated downregulation results in an increase in the cell surface levels of the A_2A_-receptor. This can also be achieved by several inhibitors of HSP90, by kifunensine (an inhibitor of the mannosidase required for ER-associated degradation), by proteasome inhibitors [52] or by overexpressing USP4, a deubiquinating enzyme, which interacts with the C-terminus of the A_2A_-receptor [51]. It can be argued that these findings are specific to the A_2A_-receptor: however, several crucial observations can also be recapitulated with the A_1_-adenosine receptor and folding-deficient mutants thereof [56]. Finally, it is worth mentioning that the receptors, which—as a consequence of these manipulations—reach the cell surface, are fully functional, i.e., they bind ligands and the enhanced expression levels translate in augmented cellular responses to agonists [51,52,56].

More importantly, a very similar mechanism operates on the completely unrelated SERT (SLC6A4), although—by contrast with the A_2A_-receptor—SERT is assisted by HSP90β rather than HSP90α: in the endoplasmic reticulum, HSP70-1A engages the proximal segment of the C-terminus adjacent to TM12. This interaction can be visualized by FRET (fluorescence resonance energy microscopy) microscopy and is limited to nascent SERT in the ER. SERT, which resides at the cell surface, does not interact with HSP70-1A [57]. Two factors may account for this spatially restricted interaction: the binding site for HSP70-1A is no longer accessible (i) in the folded state—i.e., when the C-terminus has been correctly positioned [44]—and (ii) because the thickness of the bilayer increases progressively in the secretory pathway [58,59]; thus, the segment adjacent to TM12 is available for binding of HSP70-1A to nascent SERT in the thin bilayer of the ER but access may be sterically impeded was the lipid bilayer expands in thickness in subsequent compartments. Components of the heat-shock protein relay are retrieved in abundant amounts in complex with folding-deficient mutants of SERT. The relative abundances of HSP70-1A and of HSP90β in these complexes are inversely related and reflect the severity of the folding defect: the more severe the folding-deficient phenotype the more HSP70-1A is found in complex with the SERT mutant. Both, siRNA-induced depletion of HSP70-1A and of HSP90β and their inhibition by small molecules restore cell surface expression of functional transporters in some but not all folding-deficient mutants [57]. Finally, pharmacochaperoning of folding-deficient mutants of SERT [57] and DAT [60,61] by noribogaine results in the release of the heat-shock protein relay.

Based on these observations, it is justified to argue that equivalent principles operate in very distinct polytopic membrane proteins. Hence, it is likely that a heat-shock protein relay, which monitors the state of the C-terminus to gauge progress in the folding trajectory, is of relevance to many polytopic membrane proteins. In fact, several G protein-coupled receptors have been shown to be assisted by isoforms of HSP70/HSC70 and or HSP90, including the α_2C_ adrenergic receptor [62], the melanocortin receptor-4 [63], the prostaglandin D2 receptor [64], the lysophosphatidic acid receptor-1 [65] and the β_2_-adrenergic receptor [66]. Similarly, folding of the Na^+^/Cl^−^-symporter (SLC12A3) is monitored by a cytoplasmic heat-shock protein relay [67]. The number of HSP70 (DNAK) family members is limited, i.e., there are only 11 isoforms in the human genome, which act in various cellular compartments [68]; substrate recognition is—in many instances—driven by HSP40/DNAJ family members, which are substantially more numerous, i.e., there are 41 human isoforms [68]. Currently, insights are limited, into how and which HSP40 isoforms are recruited to folding intermediates on the cytosolic side. However, there are a few examples of HSP40 isoforms that are specifically recruited to folding intermediates: ER-resident rhodopsin is recognized by HSJ1a and HSJ1b [69]; HSJ1b also targets the melanocortin receptor-4 [63]; DRiP78 (DnaJ homolog C14) interacts with the C-terminus of the D_1_-receptor [70] and of the A_1_-adenosine receptor [10]; DNAJA1/HSP40 operates on the Na^+^/Cl^−^-symporter/SLC12A3 [67]. DNAJA1 is also retrieved in complex with SERT [71]. At the very least, these findings are consistent with a model, which posits that a cytoplasmic heat-shock protein relay operates during the folding trajectory of ER-resident polytopic membrane proteins (Figure 3). In this model [37,72], the heat-shock protein relay function as a gatekeeper: ER export can only be initiated, if the heat-shock protein is released from the C-terminus and hence the protein is licensed for ER export, because the C-terminus becomes accessible for the COPII (coatomer protein complex II)-machinery. In SLC6 transporters, the binding site for the cognate cargo receptor SEC24 isoform (SEC24C or SEC24D) resides in the C-terminus adjacent to the HSP70 binding site [73,74,75]. This arrangement ensures that client membrane proteins can only be exported after they have reached the fully folded state. It is worth noting, though, that steric hindrance by the heat-shock protein relay does not require the SEC24 binding site be adjacent to the HSP70 binding site: because of the large size of the HSP90 complex and because of the annular arrangement of the transmembrane segments in polytopic membrane proteins, access of SEC24 is likely to be precluded regardless of whether the binding site is in an intracellular loop [76] or at the N-terminus [77]. We stress that this model is a simplified version, because additional chaperones [66] and gatekeepers [78,79] can monitor the folding trajectory and the assembly of oligomeric complexes prior to ER export. PRAF2 (prenylated Rab acceptor family 2) is a case in point: PRAF2 binds to the C-terminus of the GABA_B_-receptor-1 via an arginine-based motif (RSRR conforming to an RXR-retention motif) and a preceding di-leucine motif; PRAF2-release is driven by the association of GABA_B_-receptor-1 with the GABA_B_-receptor-2 [78,79]. Thus, assembly of the heterodimer and its subsequent ER export is under the control of PRAF2. This arrangement presumably allows for monitoring of the folding trajectory of the GABA_B_-receptor-2, because the heterodimer is only likely to form after the GABAB-receptor-2 has reached its stable fold. Finally, the SEC24 binding site can also be supplied in *trans* by an associated escort protein [80], which is likely to be recruited after completion of folding.

## 3. Remedying Folding Deficiency: Scaffolding vs. Relaxing Quality Control

The energy landscape of protein folding is rugged [55]: packing individual helices results in a drop in energy and thus produces local minima in the energy landscape, but rearranging these helices may destabilize interactions, which have already been formed. Thermal motion aggravates the problem. Unsurprisingly, in many instances, lowering the temperature increases the probability of productive folding and rescues folding-deficient variants. This has also been documented for GPCRs [81,82]. In practice, this approach cannot be pursued to remedy a folding disease unless the misfolded protein is expressed in testis [83]. Specific ligands are thought to act as scaffolds; by binding to folding intermediates, they allow the folding trajectory to move forward. G protein-coupled receptors have an othosteric binding site, where the cognate (endogenous) agonist(s) and antagonists bind. In addition, G protein-coupled receptors have several binding sites for allosteric activators or inhibitors [84]. Pharmacochaperoning by allosteric ligands has not yet been explored to the same extent as that of orthosteric ligands: it worth noting that allosteric ligands capable of rescuing misfolded GPCRs have been discovered for the calcium sensing receptor [85], the receptors for FSH/follicle stimulating hormone [86] and LH/luteinizing hormone [87] and the wnt-receptor frizzled-4 [88]. In all these instances, the cognate ligand is known to bind to an N-terminal domain rather than within the hydrophobic core. Thus, it is conceivable that these allosteric pharmacochaperones bind to the hydrophobic core within the region, where the orthosteric binding site lies in rhodopsin-like GPCRs. In fact, many folding-deficient G protein-coupled receptor mutants can be rescued by treating the cells with orthosteric antagonists [15]. Cell-permeable agonists also work [10]. However, their therapeutic potential is limited, because in vivo their efficacy as pharmacochaperones is compounded by agonist-induced desensitization of the receptor. The pharmacochaperoning action of orthosteric ligands relies—at least in part—on the presence of proteinaceuos chaperones heat-shock proteins. This conclusion is based on the following observations: fully functional (i.e., ligand- and G protein-binding competent) GPCRs can be expressed in *E. coli* [89,90] including the A_1_-adenosine receptor [91]. However, antagonists fail to rescue folding-deficient mutants of the A_1_-receptor, if these are expressed in *E. coli*, although in mammalian cells these antagonists effectively pharmacochaperone the very same receptors [10]. 

Pharmacochaperoning folding-deficient SLC6 transporter mutants is less straightforward: typical inhibitors and substrates fail to restore their export from the ER and their cell surface localization [43,92]. The failure of inhibitors can be rationalized by taking into account that inhibitors bind to the outward facing conformation, which requires the presence of Na^+^ ions. However, there are not appreciable levels of Na^+^ in the ER lumen [37], the topological equivalent to the extracellular face of the plasma membrane. Hence, inhibitors cannot bind to ER-resident SLC6 transporters. It is also not surprising that (membrane-permeable) substrates are ineffective: substrates drive conformational transitions and are thus unlikely to stabilize a folding intermediate. The ionic composition of the ER and the resulting transmembrane gradient predicts that the folding trajectory of SLC6 transporters ought to passes through the inward-facing conformation. In fact, ibogaine and its demethlyated metabolite noribogaine, which bind to the inward-facing conformation of SERT and DAT [93,94], were the first effective pharmacochaperones to be identified [43,57,60]. Similarly, mutations, which trap SERT in the inward-facing state [95], act as second site suppressors and restore ER-export of folding-deficient SERT mutants [57]. Subsequently, additional compounds were identified, which pharmacochaperone folding-deficient DAT [92] and SERT mutants [96]. These compounds act as atypical inhibitors of the transporter, e.g., bupropion in DAT [92], or as atypical substrates, presumably because they also have a high affinity for the inward-facing state of SERT [96].

The heat-shock protein relay, which operates on the cytosolic side, is an obvious target: inhibitors of both HSP70 [97] and HSP90 [98] are being developed for the treatment of various cancers. It has long been known that relaxing the quality control in the ER can rescue folding-deficient membrane proteins: inhibition of SERCA (the sarcoplasmic-endoreticular Ca^2+^-ATPase) by thapsigargin depletes the ER of calcium and thus abrogates the activity of calnexin. This allows folding-deficient CFTR-ΔF508 to reach the cell surface [99], albeit not in quantities sufficient to be clinically relevant [100]. Similarly, inhibition of the proteasome-dependent ER-associated degradation also allows CFTR-ΔF508 to escape to the cell surface [101]. The effect of proteasomal inhibition is not restricted to CFTR-ΔF508: an increase in ER export and surface expression of functional receptors can also be seen with GPCRs [51,52,102]. Based on these observations, it appears that ER quality control is stringent and overprotective: ER quality control is programmed to err on the safe side, which leads to the elimination of functional protein molecules. Thus, relaxing ER quality control may allow for increased ER export of mutant membrane proteins without jeopardizing cellular viability. The chaperone-COPII exchange model (Figure 3) posits that a heat-shock protein relay monitors progression of SLC6 transporters through their folding trajectory. This model also predicts that inhibition of the heat-shock protein relay relaxes ER quality control. This prediction has been verified in several instances: (i) as mentioned above inhibitors of HSP90 enhance surface expression of the A_2A_-receptor [52] and of the V_2_-vasopressin receptor [103]. Similarly, ER export and cell surface expression of some folding-deficient SERT mutants [57] and disease-associated DAT mutants [61] can be restored by inhibitors of HSP90; (ii) The HSP70 inhibitor pifithrin-μ (2-phenylethynesulfonamide) is also effective at restoring the surface expression of several folding-deficient SERT [57] and DAT mutants [60,61]. Importantly, pifithrin-μ also rescues folding-defective DAT mutants in vivo: *Drosophila melanogaster*, which lack a functional DAT, are sleepless [104]. Reduced sleep length is also seen true for flies harbouring folding-deficient DAT mutants [60,61,105]. Pifithrin-μ is as effective as the pharmacochaperone noribogaine in restoring sleep, if these flies are administered the drugs via their food [60,61]. Similarly, the increase in V_2_-receptor expression, which is induced by HSP90 inhibitors, is also seen in people: hyponatraemia is a side effect that was frequently observed in cancer patients undergoing clinical trials with various HSP90 inhibitors [103]. 

Neither inhibitors of HSP90 nor HSP70 are universally effective in rescuing folding deficient mutants. This can be rationalized by taking into account the fact that their action depends on the point, at which the folding intermediates are stalled in the folding trajectory. Accordingly, when combined with the pharmacochaperone noribogaine, all possible types of interactions were observed: depending on the nature of the mutation in SERT, HSP inhibitors (i) potentiated the action of noribogaine by shifting the concentration of noribogaine to the left; (ii) they were additive by increasing the maximum effect on cell surface expression without any appreciable change in EC_50_ of noribogaine or (iii) they shifted the concentration-response curve to the right [57]. It is also worth noting that HSP70 inhibitors are not equivalent, because they target different domains in the protein [106]. Pifithrin-μ binds to the C-terminal substrate/peptide-biding domain and suppresses the association between HSP70 and some of its co-factors/co-chaperones (e.g., HSP40) [107]. In contrast, VER155008, for instance, is an adenosine derivative that mimics the action of ADP in the HSP70 cycle and thus traps the substrate–HSP70 complex [108]. Contrary to pifithin-μ, VER155008 does not rescue folding-deficient SERT mutants but increases their accumulation in the ER [57]. 

Based on these observations, it is safe to conclude that that inhibition of HSPs is a viable option to rescue folding-deficient polytopic membrane proteins, but that many different compounds will be needed to achieve relaxation of ER quality control at the stage, where a given mutant is stalled. In vivo, orthosteric ligands, which occupy the substrate binding site in a transporter or the agonist binding site, have a major limitation: while they rescue their target, they also block it. Accordingly, they must be given in a pulsatile manner: the folding defect of a GnRH-receptor can be corrected in mice by administration of a GnRH-receptor antagonist every three days [109]. Similarly, migalastat is administered to patients every other day to restore the function of lysosomal α-galactosidase in Fabry’s disease [3]. When combined with the appropriate HSP-inhibitor, the pharmacochaperoning action of the orthosteric ligand may be selectively potentiated, because the compounds act synergistically on the folding intermediates in the ER. There is at least one proof-of-concept experiment that supports this assumption: addition of pifithrin-μ substantially enhanced the ability of low doses of noribogaine to restore sleep in flies harbouring a folding-deficient DAT mutant [60].
